# Complement and macrophage crosstalk during process of angiogenesis in tumor progression

**DOI:** 10.1186/s12929-015-0151-1

**Published:** 2015-07-22

**Authors:** M. Afzal Khan, A. M. Assiri, D. C. Broering

**Affiliations:** Department Comparative Medicine, King Faisal Specialist Hospital and Research Centre, MBC 03, P.O. Box 3354, Riyadh, 11211 Kingdom of Saudi Arabia; Organ Transplant Centre, King Faisal Specialist Hospital and Research Centre, Riyadh, Kingdom of Saudi Arabia

**Keywords:** Tumor progression, Complement mediated injury, Angiogenesis, Macrophage

## Abstract

The complement system, which contains some of the most potent pro-inflammatory mediators in the tissue including the anaphylatoxins C3a and C5a are the vital parts of innate immunity. Complement activation seems to play a more critical role in tumor development, but little attention has been given to the angiogenic balance of the activated complement mediators and macrophage polarization during tumor progression. The tumor growth mainly supported by the infiltration of M2- tumor-associated macrophages, and high levels of C3a and C5a, whereas M1-macrophages contribute to immune-mediated tumor suppression. Macrophages express a cognate receptors for both C3a and C5a on their cell surface, and specific binding of C3a and C5a affects the functional modulation and angiogenic properties. Activation of complement mediators induce angiogenesis, favors an immunosuppressive microenvironment, and activate cancer-associated signaling pathways to assist chronic inflammation. In this review manuscript, we highlighted the specific roles of complement activation and macrophage polarization during uncontrolled angiogenesis in tumor progression, and therefore blocking of complement mediators would be an alternative therapeutic option for treating cancer.

## Introduction

The complement system has been primarily considered as an effector of innate immunity with the ability to antibody-mediated disposal of foreign particles, apoptotic clearance, and actively maintain the phase of immune surveillance in different inflammatory states [1–4]. The complement activation play a dual role in tumor development, and it has been reported that it can control tumor activities through acute inflammation, immunostimulation, lysis, opsonization and chemotaxis [5]. In contrast, the complement activation also support chronic inflammation, promote an immunosuppressive microenvironment, induce angiogenesis, and activate cancer-related signaling pathways during tumor progression [6]. Process of angiogenesis is firmly regulated by the balance of two sets of counteracting mediators known as– angiogenic activators and inhibitors [7]. The stage of 'angiogenic-switch' determines the time of initiation of succeeding angiogenic phase and a physiological up-regulation of angiogenic stimulators than angiogenic inhibitors, as in the case of wound healing, which will turn-on angiogenic switch to speed up the process of normal angiogenesis [8, 9]. During the process of normal angiogenesis, each step is tightly remained under the control of different immune regulators but in some pathological cases, the 'angiogenic-switch' remains in the active state, which leads to uncontrolled angiogenesis in many physiological disorders including tumor progression [10]. Malignant cells are able to maintain a balance between complement activation and inhibition thereby manipulates most advantages of complement initiation without suffering its destructive effects [11, 12]. In this perspective, inhibition or blocking of complement mediators would be a potential therapeutic option for treating cancer [12–14]. This review further highlights how cancer cells manipulates complement mechanism to protect their proliferation, and survival by recruiting tumor-associated macrophages (TAMs) and facilitates tumor progression.

A. How do tumor cell skip anti-tumor effects of inflammation?

**Fig. 1:**
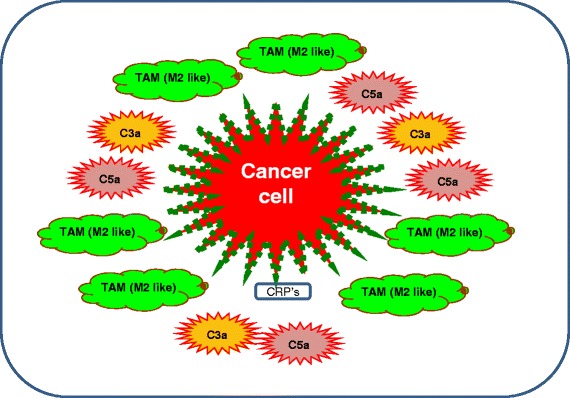
Mechanism of cancer cell protection. Model illustrates the shielding mechanism orchestrated by cancer cells to protect against host effector immune responses. It shows complement resistant cancer cells are hugely infiltrated with C3a and C5a, TAMs and over-expressed complement regulatory proteins (GREEN dots)

Tumor progression has a unique property, which makes tumor cells to skip the anti-tumor effects of inflammation in order to polarize immune responses toward those effectors that facilitates tumor progression. It has been reported that cancer cells adopt a variety of cellular machineries to trick complement-mediated injury [15]. The deleterious roles of complement mediators on the eradication of tumors have not been investigated in details. However, by virtue, complement has tendency to recognize non-self-elements, and it is interesting to note that any structural manipulations in the tumor cell membranes make these cells an easy target for complement attack, and a number of clinical studies have reported an activation of complement in cancer patients [16, 17]. The activation of complement cascade release key anaphylatoxins C5a and C3a, which are potent chemo-attractants for macrophages, eosinophils, monocytes, and T cells [18, 19] and modulate complement mediated tissue injury through the release of cytokines, eicosanoids, and reactive oxygen species [18, 19]. To protect from the complement, cancer cells develop a variety of configurational strategies to combat complement-mediated damage [15]. Due to these structural manipulations, though, malignant cells are recognized by complement mediators but survive complement mediated lysis through the shielding role of complement regulatory proteins [20] (Fig. 1). This regulated complement activation provides a permanent source of complement mediators that could support the tumor growth favorable inflammatory microenvironment [21, 22], and it was shown in number of *in-vitro* cancer cell line studies that the overproduction of complement activation product C5a [5]. In addition, elevated levels of plasma C5a has been reported in Lung cancer [5, 23], and in other tissue specific cancers [23, 24]. Furthermore, It has been found in number of cancer models that tumor inflammatory microenvironment constitutes complement activated fragments C3, C4, and C5, Clq, and MAC [12], high levels of IL-6 [25] and TGF-β [26]. IL-6 has been shown to assist tumor progression though apoptosis inhibition, angiogenesis stimulation [27].

**Fig. 2:**
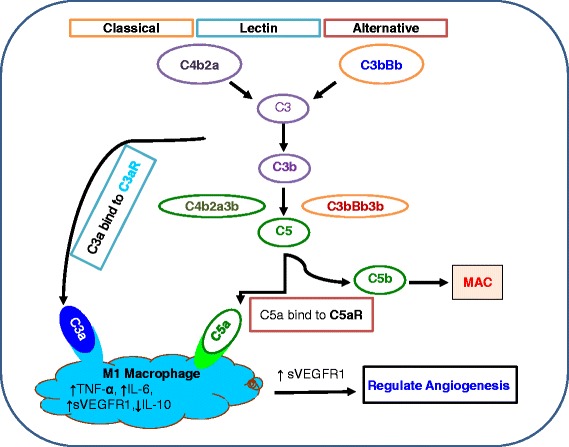
Macrophage polarization: Model illustrates M1 and M2 macrophage mediated angiogenesis in tumor development. M2 macrophages promote tumor growth through the release of TGF-β, IL-10, IL-17, IL-23, VEGF, and FGF2 required for tumor growth. In contrast, M1 macrophages suppress tumor growth through the release IFN-γ IL-12, and TNF-α

B. Complement cascade mediators:

The complement cascade consists of plasma and membrane-bound proteins, which protects against immune-mediated tissue injury in different pathogenic conditions [28]. Activation of complement cascade initiated through three different pathways: the classical pathway, the lectin pathway, and the alternative pathway. The three pathways all converge in the activation of the pivotal complement molecule C3 and generate C3 convertase [29]. This C3 convertase promotes the cleavage of C3 into C3a and C3b. After cleavage of C3, C3b molecule combines with the C3 convertase to form C4bC2aC3b complex in classical and lectin pathways, and to the formation of C3bBbC3b complex in the alternative pathway. Both C4bC2aC3b and C3bBbC3b complexes are known as C5 convertase and cleaves C5 into C5a and C5b molecules. The generated C5a can then function as a potent anaphylatoxin at the site of production while C5b participates in the assembly of the membrane attack complex (C5b-9 or MAC) [29]. Finally, MAC complex can initiate cell lysis, and in sublytic quantities can lead to cell activation (Fig. 2). The main sources of C3 in human are hepatocytes [30], but C3 is also expressed by macrophages [31], fibroblasts [32], vascular endothelium [33], astroglia [34] and adipocytes [35]. Beside of involvement in complement activation, C3 and its degradation products are able to promote phagocytosis, activate inflammatory responses against pathogens, and regulate adaptive immune, but uncontrolled activation may result in host cell damage [36]. The complement cascade plays a vital role in antimicrobial defense and the clearance of immune complexes and apoptotic cells [37]. This latter property is a recognized pathogenic factor in a wide spectrum of chronic inflammatory diseases, including rheumatoid arthritis, glomerulonephritis, atherosclerosis, asthma, and multiple sclerosis [38]. Substantial research has proven the evidence of the activated complement mediators in cellular proliferation, and diseases of chronic inflammation, which suggest a potentially deleterious role in abnormal cellular growth [18, 39]. Thus, it is not overwhelming that signals for complement-mediated disease pathogenesis has centered mainly on the dysfunctional immunity caused by the absence, alteration, or over activation of complement proteins [39]. It has been reported that complement proteins mediate cellular turnover, growth, and regeneration, including bone marrow stem cell engraftment, bone and cartilage development, neurogenesis, synaptogenesis, white matter healing, and regeneration of the liver, limb, and lens [12, 40, 41].

**Fig. 3:**
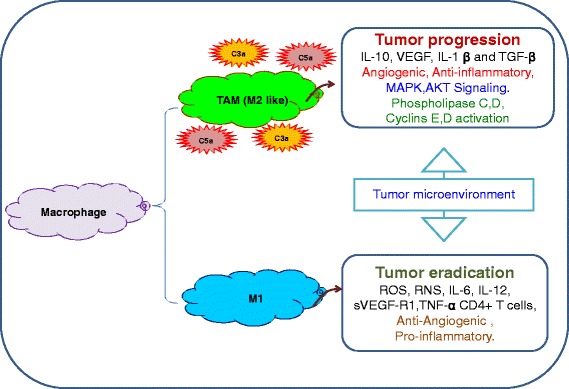
Regulation of angiogenesis. Model illustrates macrophage-mediated regulation of angiogenesis: This explains how complement mediators regulate the phase of angiogenesis under normal physiological conditions

C. Macrophages during tumor progression:

Macrophages are an important component of the innate immune system, they are characterized by the expression of number of unique surface markers including CD14, CD11b, CD11c, F4/80, MAC-1/MAC-3, CD163, and CD68 [42, 43]. It has been found in number of tumor models that macrophages support cancer cells proliferation and survival [44]. Macrophage release TNF-α and activate NF-kB, which turns-on proteins that stop apoptosis, and promote cell proliferation [45–47]. Macrophages maintain essentials of tissue remodeling, inflammation, and immunity, include endocytosis of foreign and necrotic debris, cytotoxicity, and secretion of more than different cytokines [48–50]. However, depending on their activation, macrophages are able to secrete growth factors, prostaglandins, interferon, elastase, plasminogen activator, and collagenase, and complement components C3 and C5 [51]. Macrophages have classically activated M1 and alternatively activated M2 subtypes, and the differentiation in to respective subtype is mediated through the release of cytokines and growth factors present in the inflammatory microenvironment [52–54]. The development of M1 macrophages stimulated through IFN-γ and TNF-α [55], whereas, the M2 macrophages are polarized by IL-4, IL-13 [54]. As mentioned, the immunological effects of both M1 and M2 subtypes are regulated through the myriad of cytokines. It has been reported that IFN-γ stimulated M1 macrophages elevate the production of IL-12 and IL-23, and low IL-10 [50, 56]. However, M2 macrophages favor over production of IL-10 and low levels of IL-12 and IL-23 [56, 57]. M1 macrophage secreted IL-12 promotes the differentiation of Th1 cells, which can improve antigen phagocytosis [48], while IL-23 triggers the Th17 cells proliferation and release of IL-17, which modulates inflammatory autoimmune pathologies [58, 59]. On the other side, the M2 macrophage secreted IL-10 favor the production of IL-4 and IL-13 by Th2 cells [60]. The M1 macrophage derived chemokines are also critical for killing intercellular pathogens, whereas the M2 macrophage derived chemokines promote the recruitment of the leukocytes involved in tissue repair and remodeling process [54]. It has been reported that macrophage polarization occurs during onset of tumor progression and it changes from classically activated M1 to alternatively activated M2 like Tumor-Associated Macrophages (TAMs) [54]. Depending on the physiological and pathological conditions, macrophages undergo differentiation into M1 and M2 phenotypes and both play a key role in maintaining tumor growth [61]. M1 macrophages are pro-inflammatory in function, they secrete IFN-γ, IL-12 and TNF-α, and therefore effectively suppress tumor growth [62, 63] while M2 like TAMs secrete TGF-β, IL-10, IL-17, IL-23, VEGF, and FGF2 [62], promote angiogenesis and tumor progression (Fig. 3). M2 TAMs produce VEGF, FGF chemokines IL-17, IL-23, or TGF-β that contribute to stimulation of vascular endothelial cell proliferation, release of MMPs, which degrade the vascular basement membrane and induce sprouting, migration of endothelial cells into the tumor and this process further leads to tube formation and maturation of new microvessel. [62]. M2 TAMs promotes the proliferation of tumor cells directly by secreting growth factors, and participate in tumor progression by acting on endothelial cells, and thus promoting the neovascularization of the tumor [49, 62, 64]. M2 TAMs are the key player during angiogenesis and promote each step of the angiogenesis cascade [56, 65, 66]. The angiogenic phenotype of macrophages is in part defined by their ability to secrete molecules that promote or inhibit angiogenesis [54, 56, 65–67]. During tumor progression, M2 TAMs are migrated to tumors under the influence of various cytokines and growth factors secreted by cancer and stromal cells. However, it has been shown that M1 macrophages express IFN-γ, IL-1, and IL-6, which prime T-cell towards anti-tumor type-1 inflammatory response [68]. However, in most tumors such as breast, prostate, ovarian, cervical, lung carcinoma, and cutaneous melanoma, M2 TAMs are considered to be anti-inflammatory and correlated with a poor prognosis [56, 69]. M2 TAMs mostly occupy hypoxic regions of tumors, and express more proangiogenic genes, such as VEGF, pFGF, CXCL8, and glycolytic enzymes, through the transcription factors HIF-1α and HIF-2 [70, 71]. Most TAMs display M2 associated molecules, which includes IL-10 and MGL1, as well as CCL2, CCL5, CXCL9, CXCL10, and CXCL16, and heat shock proteins [72, 73]. In addition, the tumor microenvironment, which includes IL-4, IL-13, TGF-β, and IL-10 further support the adoption of an M2 phenotype [54]. M2 TAMs are associated with different stages of tumor development, which includes tumor progression, angiogenesis, uncontrolled growth, actual metastasis, immunosuppression, matrix deposition, and tissue remodeling [56, 74]. It has been reported that M2 TAMs are able to modulate and induce neovascularization and support functions. In addition, activated TAMs release growth factors (VEGF and PDGF), cytokines (TGF-β), proteases, and chemokines, which promote angiogenesis in many tumors, such as gliomas, squamous cell carcinomas of the esophagus, and breast, bladder, and prostate carcinomas [56, 65]. Moreover, TAM-secreted matrix metalloproteases (MMP-1, MMP-2, MMP-3, MMP-9, and MMP-12), and plasmin are also support angiogenesis [75]. In addition, MMP-9 support angiogenesis through the release of VEGF signaling [76]. TAMs preferentially accumulate in hypoxic and necrotic regions within the tumors and become M2-angiogenic [62].

D. Complement activation during angiogenesis and in tumor progression:

The complement regulates both innate and adaptive immune systems, and more particularly during the phase of acute inflammation, complement anaphylatoxins C3a and C5a mediates changes in microvascular flow, permeability, leukocyte extravasation and migration, which contributes to tissue damage after ischemia reperfusion and in other disease models [2, 77]. The active complement proteins, C3a and C5a, control macrophage mediated angiogenesis in tissue [12, 40, 41]. C3a and C5a generated from complement cascade have potential effects on macrophage-mediated angiogenesis inhibition. Both C3a and C5a anaphylatoxins bind to their respective C3aR and C5aR on monocytes/macrophages [78] and many other cells including airway epithelium, causing an anti-angiogenic response resulted in an increased IL-6, TNF-α, sVEGFR1, and decreased IL-10 mRNA. Increased sVEGFR1 secretion from monocytes/macrophages inhibits angiogenesis in different disease models [40, 41] (Fig. 2). It has been well established in transplants model that C3^−/−^ recipients mice exhibited more dilated and leaky microvessels as compare to the control allografts [2, 77]. In addition, the latest research outcomes in mouse model of retinopathy reveals the importance of complement in regulating increased neovascularization in C3^−/−^ mice compared with control groups [79]. Along with C3 role in neovascularization, C5a also play a crucial role in angiogenesis, and increased neovascularization was observed in C5aR^−/−^ mice compared with control group [79]. C5aR antagonist supported the process of neovascularization, whereas treatment with C5a agonist reduced neovascularization in retinopathy model, confirming the inhibitory role of C5a and C5aR in this process [79]. Moreover, treatment of C3^−/−^ with C3a and C5a reduced the enhanced neovascularization effect observed in C3^−/−^ mice, suggesting that C3a and C5a are inhibitory effectors of pathologic hypoxia-driven retinal neovascularization [79].

Complement proteins have been reported to facilitate tumor progression through the dysregulation of mitogenic signaling pathways, continuous cellular proliferation, angiogenesis, insensitivity to apoptosis, invasion and migration, and escape from immunosurveillance [12, 14, 80]. During the phase of tumor progression, complement anaphylatoxins C3a and C5a increase mitogenic signaling pathways. The cell inducing abilities of the C3a and C5a have been reported [81] and also revealed the involvement in several other signal transduction pathways with known links to neoplastic progression (Fig. 3). In addition, complement receptors (C3aR1) and (C5aR1) are coupled to G-proteins and mostly present in M2 macrophages, airway epithelium, and parenchymal vessels. Number of research findings have shown that the membrane attack complex, which is one of the main terminal product of complement cascade, also activates the cell cycle and oncogenic pathways [82, 83]. It is well known and established that under hypoxic conditions, progression from a small population of neoplastic cells to a clinically significant mass requires creation of new microvessels to perfuse the newly formed malignant tissue structure [84]. Moreover, number of studies has revealed that the extent of tumor vessels formation suggested a direct link between increased angiogenesis and tumor aggressiveness, can predict the growth rate and progression of the disease [85]. Activation of complement cascade has been examined through *in-vitro* studies of cancer cell lines and I was found that Lung cancer cells are able to produce C5a more efficiently than do non-malignant bronchial epithelial cells but the mechanism is still not yet explained [5]. The classical pathway has been recognized as the major contributor to complement activation on subcutaneously inoculated TC-1 cervical cancer cells [13] and in two neuroblastoma cell lines in vitro [17]. In the case of primary tumors, Lucas et al. [86] have reported that a tumor-specific immune activation occurs in papillary thyroid carcinomas, with activation of the classical pathway. In addition, role of classical pathway has also been reported in follicular, MALT lymphomas [87], and in patients with chronic lymphocytic leukemia [88]. In contrast, the results of other studies has demonstrated that lymphoma and myeloma cells activate the alternative pathway of the complement cascade [89]. Furthermore, both the alternative and the classical cascade has also been reported in some cases [90, 91], while the lectin pathway has been found to be significantly activated in colorectal cancer patients. The immuno regulatory functions of C3a and C5a and their receptors (CR) has been characterized in cancer models, and it was found that number of myeloid-derived innate immune cells express C3aR and C5aR, including monocytes, macrophages, DCs, neutrophils, basophils, T cells, mast cells, and eosinophils [92–94]. Through, its influence on innate immune cells including DCs and macrophages, C3a similarly regulates the T cell response, especially the determination of Th1 cells [14].

## Conclusions

The complement system help innate immune attack against cancer cells through cytotoxic and lytic effects but number of studies are revealing that the complement cascade enables a remarkable array of proliferative events [20]. Both complement and macrophages interact closely to maintain process of angiogenesis, and modulate the major features of carcinogenesis, including dysregulation of mitogenic signaling pathways, sustained cellular proliferation, angiogenesis, and insensitivity to apoptosis, invasion, metastasis, and escape from immuno-surveillance. Macrophages also tune inflammation and adaptive immunity, promote cell proliferation by release of number of growth factors, ornithine, and polyamines. They also take active role in promoting angiogenesis, tissue remodeling, and tissue repair [66, 95, 96]. M2 macrophages acts as a tumor promoters at distinct phases of malignant progression of gastric, mammary [97], lung [98], and liver carcinomas [99]. Macrophages express receptors for activated C3 and C5, respond to activated C3a and C5a mediators at the site of local inflammation, and maintain angiogenesis in tissue [79]. There are significant research evidences, which support the contribution of complement activation to tumor progression [22, 24, 100]. During tumor progression, tumor cells undergo genetic and epigenetic manipulations that modulate their malignant growth [5, 101, 102]. Due to these manipulations, the complement system can differentiate and recognize tumor cells from non-malignant cells [29, 103, 104], which protect cancer cells from deleterious effects of complement mediators [105]. It has been demonstrated in mouse model of cervical cancer that the generation of C5a in the tumor microenvironment promotes tumor progression through the recruitment of myeloid-derived suppressor cells and the generation of an immunosuppressive microenvironment [106]. However, in lung carcinogenesis model, C5a play a key role as a mediator in the regulation of cancer growth [5], and targeted inhibition of the C5a bindings to its receptors could inhibit the microenvironment without diminishing the protective effects of complement activation [107]. In addition to its anti-inflammatory role, complement play a crucial role in several transduction pathways involved in tumor progression. C5a is a key regulator of complement mediated inflammatory response during tumor progression [18]. The therapeutic blockade of the complement system to suppress tumor progression has been investigated by antagonizing the C5aR receptor [13], and it was suggested that tumor cells evade lysis by the use of protective mechanism that limits the formation of functional MAC pores [108]. In addition, It has been demonstrated that MAC activates cancer-associated signaling pathways through MAPK, phosphatidylinositol 3-kinase, Ras, and p70 S6 kinase [82, 109, 110], and inhibit apoptosis cancer cells by blocking FLIP, caspase-8 [111].

These findings reflects that cancer cells utilize complement modulation as a shield to protect itself from surrounding effector molecules, and thereby favors tumor progression. This unique adoptability of cancer cells uncover the key importance of targeted complement inhibition to restrict tumor growth [105, 112]. A number of studies are targeting the potential role for complement-mediated therapeutics options to rescue tissue from tumor progression. There is particular interest in the applications of monoclonal antibodies against the soluble and membrane-bound complement inhibitors reported by different tumors, an approach believed to enhance antitumor complement-dependent cytotoxicity and antibody-dependent cell-mediated cytotoxicity. However, the increasingly interest of complement interaction with tumor cells demands further information, and suggest possibility that anti-complement strategies [2, 77] may offer an entirely new means of fighting number of complement mediated diseases including cancer and tumor progressions. This review uncover the cross-talk between complement proteins and macrophages on tumor progression and future of complement as a potential therapeutic target to rescue tissue from tumor progression.

## Abbreviations

C3AR1: Complement component 3a Receptor 1
C5AR1: Complement component 5a Receptor 1
CR: Complement receptor
ECM: Extra cellular matrix
FGF: Fibroblast growth factor
MAC-1 & -3: Macrophage-1 & -3 antigen
MAC: Membrane attack complex
MMPs: Matrix metallo peptidase
MALT: Mucosa-associated lymphoid tissue
PDGF: Platelet-derived growth factor
PGD2: Prostaglandin D2
TAMs: Tumor-associated macrophages
